# An *Agrobacterium*‐delivered CRISPR/Cas9 system for targeted mutagenesis in sorghum

**DOI:** 10.1111/pbi.13229

**Published:** 2019-08-21

**Authors:** Si Nian Char, Jialu Wei, Qi Mu, Xianran Li, Zhanyuan J. Zhang, Jianming Yu, Bing Yang

**Affiliations:** ^1^ Department of Genetics, Development and Cell Biology Iowa State University Ames IA USA; ^2^ Department of Agronomy Iowa State University Ames IA USA; ^3^ Division of Plant Sciences University of Missouri Columbia MO USA; ^4^ Donald Danforth Plant Science Center St. Louis MO USA

**Keywords:** CRISPR/Cas9, sorghum, mutagenesis, *GA2ox5*, *FT*, flowering time

To the Editor:

Clustered regularly interspaced short palindromic repeat/CRISPR‐associated Cas9 (CRISPR/Cas9) systems of bacteria and archaea have engineered for genome editing in eukaryotic genomes. In such CRISPR/Cas9 system, CRISPR RNA (crRNA) and trans‐activating CRISPR RNA (tracrRNA) were engineered into a simplified single guide RNA (sgRNA). Cas9 and sgRNA form a complex that scans through genome for the protospacer adjacent motif (PAM) sequence (predominantly 5′‐NGG‐3′) and for the sequence (ca. 18–20 nucleotides) complementary to the sgRNA, leading to double‐stranded DNA breaks (DSBs) that are exploited for site‐specific DNA alterations (Jinek *et al*., 2012).

Sorghum is the fifth most important cereal crop across the globe. Genome‐editing platform in sorghum was lagging behind other cereal crop plants due to its low stable transformation efficiency. Cas9/sgRNA activity was demonstrated for mutagenesis in sorghum callus cells derived from immature embryos using reporter gene encoding for red fluorescence protein (Jiang *et al*., 2013). One recent study has reported the application of CRISPR/Cas9 that targeted the centromere‐specific histone 3 (*SbCENH3*) gene, while the inheritance of the CRISPR‐induced mutations has yet to be demonstrated (Che *et al*., 2018). More recently, targeted mutagenesis of the *k1C* gene family encoding for α‐kafirins with a single guide RNA to target the conserved region of multiple *k1C* genes led to increased protein digestibility and lysine content in sorghum grain (Li *et al*., 2018a). Here, we report the development of a CRISPR/Cas9 system tailored for *Agrobacterium*‐mediated transfer and validation of its efficiency for targeted mutagenesis in two endogenous genes *SbFT* (Sb10G045100) and *SbGA2ox5* (Sb09G230800). The CRISPR‐induced mutations were able to pass on to the T_1_ generation. The continuing activity of Cas9/sgRNA can lead to more site‐specific mutations in next generation as long as the Cas9/sgRNA transgene is still present in the sorghum plants. The *SbFT* mutant plants exhibit significant difference in flowering time.

We first adapted a schematic workflow and cloning strategy for sorghum CRISPR/Cas9 system (Figure 1a). To assemble the CRISPR/Cas9 system for sorghum mutagenesis through *Agrobacterium*‐mediated gene transfer, three vectors were designed and constructed. An intermediate vector (pgRNA1) was used for constructing guide RNA genes through sequentially inserting two spacer sequences each fused with the guide RNA scaffold. Another pENTR4‐derived intermediate vector (pCas9:GFP) contains a *Cas9* gene directed by the maize *ubiquitin 1* gene promoter and a cloning site (*BssH*II) for accepting the sgRNA cassette. The destination vector was adapted from a binary vector (pTF101.1), suitable for sorghum transformation (Paz *et al*., 2004), to accept the Cas9/sgRNA cassette through a Gateway recombination reaction, resulting in a single *Agrobacterium*‐borne Cas9/sgRNA construct. The pTF101.1‐derived sorghum CRISPR/Cas9 construct also has the *bar* gene that is driven by 2× CaMV 35S promoter for selection of glufosinate or bialaphos‐resistant callus cells and plants in transformation process. The genotype P898012 of sorghum was transformed by following a protocol as described previously (Do *et al*., 2016).

**Figure 1:**
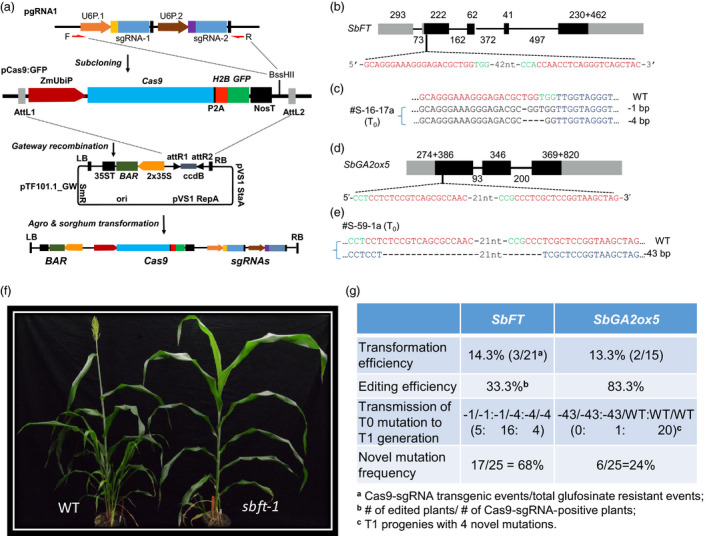
(a) Cloning strategy to construct sorghum CRISPR/Cas9. pgRNA1, a guide RNA cloning vector that contains *Btg* *Z*I and *Bsa*I sites for sequential insertions of two double‐stranded oligos for spacer sequences of two sgRNAs. Two different rice U6 promoters are used to drive each of the sgRNAs. The sgRNA cassette was PCR‐amplified with primers F and R; the amplicon is cloned into pCas9:GFP vector at *Bss* *H*II site through Gibson assembly. Through Gateway recombination, the Cas9 and sgRNA expression cassettes are mobilized into plant expression vector pTF101.1_GW for *Agrobacterium‐*mediated sorghum transformation. (b) Gene structure of *SbFT* on chromosome 10 with sgRNA target sites (in red) and PAM sequences (in green). Forty‐two nucleotides (42nt) indicate sequence not shown between two target sites. Bars in black colour denote coding regions, and bars in grey colour represent untranslated regions. Numbers indicate the nucleotides in exons (above bars) and introns (below lines). (c) Biallelic mutants (1‐bp and 4‐bp deletion) at the first gRNA target site detected in a T_0_ plant #S‐16‐17a. The nucleotide changes were indicated (dashed lines for deletion and WT for wild type). Dots represent nucleotides not shown. (d) Gene structure of *SbGA2ox5* on chromosome 9 with sgRNA target sequences (in red colour) and PAM sequences (in green). Number 21 indicates nucleotides between two target sites not shown. Bars (in solid black) denote coding regions and bars (in grey colour) represent the 5′ and 3′ UTR, respectively. Numbers of nucleotides are denoted as exons (above bars) and introns (below lines). (e) The mono‐allelic mutation (43‐bp deletion) occurring across both gRNA target sites detected in a T_0_ plant #S‐59‐1a. The nucleotide changes were indicated (dashed lines for deletion and WT for wild type). Dots represent nucleotides not shown. (f) Flowering time was affected by CRISPR‐induced mutation. Morphological characterization of wild type and mutants in the T_1_ generation. The frameshift mutation created by CRISPR/Cas9 shows the mutant plant (*sbft‐1*,* n *=* *16, right side) flowered 10 days later on average compared with wild‐type plant (*n *=* *34, left side). (g) Summary of transformation efficiency, CRISPR/Cas9 induced gene‐editing efficiency, transmission of T_0_ mutation to T_1_ generation and novel mutations happen in T_1_ progeny of *SbFT* and *SbGA2ox5*.

In this study, two candidates, based on literatures and bioinformatic analyses, as the genes underlying quantitative trait loci (QTLs) for flowering time and plant height, were selected for targeted mutagenesis. The target region of *SbFT* is located at the first exon, 13‐bp downstream of the translation start site (Figure 1b). Similarly, target region of *SbGA2ox5* is also located in exon one, 329‐bp downstream of the translation start site (Figure 1d). For pCas9‐gSbFT, three out of twenty‐one transformation events (multiple plants/event) were transgene positive at a frequency of 14.3% (Figure 1g), while two out of fifteen events were transgene positive at a rate of 13.3% for pCas9‐gSbGA2ox5 (Figure 1g). The high number of non‐transgenic escapes might be due to the lower concentration of glufosinate applied during the *in vitro* selection processes of callus induction and plant regeneration.

Genotyping three pCas9‐gSbFT‐positive plants, each from a transformation event, identified one plant (S‐16‐17a) had a genotype of biallelic mutants, 1‐bp and 4‐bp deletions (‐1bp/‐4 bp), at the first target site (Figure 1c), whereas plants S‐66‐8b and S‐66‐9a both had the wild‐type allele of *SbFT*. Among the two transformation events positive for pCas9‐gSbGA2ox5, all five plants (S‐59‐1a, ‐1b, ‐1c, ‐1d and ‐1e) of S‐59‐1 possessed the identical mono‐allelic mutation of 43‐bp deletion (Figure 1e); no mutation was detected in S‐59‐6a. Our results showed that the site‐specific mutations at the *SbFT* and *SbGA2ox5* loci in sorghum genotype P898012 were induced by the two CRISPR/Cas9 constructs.

Twenty‐five progeny plants from S‐16‐17a were used to analyse for the transmission of mutations. We observed a Mendelian segregation ratio of 1:2:1 for ‐1/‐1, ‐1/‐4 and ‐4/‐4 genotypes along the 25 T_1_ progeny plants (*P *=* *0.36, df = 2, χ^2^ = 2.04, chi‐square test; Figure 1g). For *SbGA2ox5*, similar analysis indicated that the mono‐allelic 43‐bp deletion in S‐59‐1a was passed on to only one T_1_ progeny plant (Figure 1g). Additionally, new heterozygous mutations occurred in some progeny plants in S‐59‐1a (Figure 1g). It is worthwhile to notice that recent studies indicate that *SbGA2ox5* is not the underlying gene for the corresponding plant height QTL, but unexpectedly, we failed to detect any homozygous 43‐bp deletion in the T_1_ progenies, suggesting a potential lethality of homozygous knockout of *SbGA2ox5* (Hilley *et al*., 2016; Yamaguchi *et al*., 2016). Coincidently, no *SbGA2ox5* homozygous knockout mutants were detected in the 25 T_1_ progeny plants from event S‐59‐6a described below. All the novel discovered mutations are mono‐allelic mutations including + 1/WT, ‐50/WT, ‐1/WT and ‐15/WT. Further verification of the relevance of the knockout genotype to the biological significance is needed.

To determine whether CRISPR/Cas9 expression continuously induced new mutations in T_1_ generation, twenty‐five plants derived from self‐pollinated S‐66‐9a (containing sgRNA for *SbFT*) and twenty‐five plants from event S‐59‐6a (containing sgRNA for *SbGA2ox5*) were genotyped. These two lines were chosen because they carried the CRISPR transgenes and also possessed the unedited target genes in T_0_ generation. The progeny plants were characterized for newly induced mutations at the CRISPR target sites. The presence of Cas9 and sgRNA in the gametes of sorghum plants during meiosis or in zygotes might result in additional mutations in the unedited alleles. Sanger sequencing analyses from the PCR amplicons of relevant regions showed that novel mutations were induced in both *SbFT* and *SbGA2ox5* genes. Seventeen out of twenty‐five plants (68%) with novel mutations were detected in *SbFT,* and six out of twenty‐five (24%) with newly induced mutations were identified in *SbGA2ox5* genomic target site (Figure 1g).

Expression profile of *SbFT* indicates that it is the most plausible florigen‐coding gene in sorghum and the gene underlies the flowering time QTL (Li *et al*., 2018b; Wolabu *et al*., 2016). The T_1_ plants from S‐16‐17a and S‐66‐9a events were grown in the greenhouse, and we planted them on two different dates. For the first planting date, plants containing knockout mutations flowered 8 days later than wild‐type plants on average (*P *=* *0.020, df = 13, two‐sample *t* test). The flowering time difference for the second planting date between edited plants and wild types was 10 days (*P *<* *0.001, df = 33, two‐sample *t* test) (Figure 1f). These frameshift mutations induced by CRISPR/Cas9 verified that Sobic.010G045100 is the gene underlying the detected flowering time QTL. The results also support our previous conclusion that the PIF/Harbinger insertion at 4 kb upstream in P898012 appeared to be the functional polymorphism disrupting the expression of *SbFT* (Li *et al*., 2018b).

To address the issue of potential off‐targets with gRNAs targeting *SbFT* and *SbGA2ox5*, we searched the potential sequence homology between on‐target sequences (both spacer and PAM) of gRNAs and the sorghum Tx623 reference genome. For the *SbFT* gRNAs, the closest match to the 21‐nt targeting site is only 15‐nt with PAM sequence missing, suggesting a less likely off‐target site in the transgenic sorghum plants. The similar results were found in the second target site. For *SbGA2ox5*, the first 19‐nt on‐target sequence matches one potential off‐target sites of 16 nt which misses the PAM sequence, an essential component for Cas9 recognition. The second 20‐nt target site of *SbGA2ox5* is unique since there is only one 15‐nt hit also with PAM sequence missing. Therefore, the potential off‐target effects in the sorghum CRISPR/Cas9 system can be greatly reduced by carefully choosing and designing gRNAs for the target genes.

## Authors’ contribution

SNC, XL, JY and BY designed the experiment; SNC, JW and QM performed the experiments; ZJZ supervised sorghum transformation; SNC, JW, QM, XL, ZJZ, JY and BY analysed the data and wrote the manuscript.

## Conflict of interest

The authors declare no conflict of interest.
